# Reinforcement Learning-Based Tracking Control of USVs in Varying Operational Conditions

**DOI:** 10.3389/frobt.2020.00032

**Published:** 2020-03-20

**Authors:** Andreas B. Martinsen, Anastasios M. Lekkas, Sébastien Gros, Jon Arne Glomsrud, Tom Arne Pedersen

**Affiliations:** ^1^Department of Engineering Cybernetics, Norwegian University of Science and Technology, Trondheim, Norway; ^2^Centre for Autonomous Marine Operations and Systems, Norwegian University of Science and Technology, Trondheim, Norway; ^3^Digital Assurance Program, Group Technology and Research, DNV GL, Trondheim, Norway

**Keywords:** reinforcement learning, trajectory tracking, optimal control, model-based adaptive control, approximate dynamic programming (ADP), dynamic positioning (DP), autonomous ships, system identification

## Abstract

We present a reinforcement learning-based (RL) control scheme for trajectory tracking of fully-actuated surface vessels. The proposed method learns online both a model-based feedforward controller, as well an optimizing feedback policy in order to follow a desired trajectory under the influence of environmental forces. The method's efficiency is evaluated via simulations and sea trials, with the unmanned surface vehicle (USV) *ReVolt* performing three different tracking tasks: The four corner DP test, straight-path tracking and curved-path tracking. The results demonstrate the method's ability to accomplish the control objectives and a good agreement between the performance achieved in the Revolt Digital Twin and the sea trials. Finally, we include an section with considerations about assurance for RL-based methods and where our approach stands in terms of the main challenges.

## 1. Introduction

Control of marine vehicles is a challenging problem, mostly due to the unpredictable nature of the sea and the difficulty in developing accurate mathematical models to represent the varying marine vehicle dynamics. As a result, considerable research effort has been dedicated to the topic since the early 90's (Fossen, 1994), resulting in a vast literature utilizing ideas from virtually every branch of control engineering: Linear, non-linear, adaptive, intelligent, optimal, fuzzy, and stochastic control approaches, to name a few, have been developed and tested over the years, and many of their properties are well-understood (Hasegawa et al., 1989; Pettersen and Egeland, 1996; Katebi et al., 1997; Fossen, 2000; McGookin et al., 2000; Soetanto et al., 2003; Wang et al., 2015; Do, 2016). Due to the fact that the hydrodynamic coefficients, and consequently the behavior, of a marine vehicle can vary significantly in different speed regimes, a common approach has been to design controllers for specific motion control scenarios. This approach simplifies the vessel modeling process and has led to dynamic positioning (DP) and station keeping controllers for speeds close to zero, and trajectory tracking or path following (depending on whether temporal constraints are considered) controllers when a vessel is in transit mode. Naturally, the main drawback is that, when moving from one speed regime to another, controllers and/or models with different properties are needed. Two well-researched ways to achieve such performance diversity with conventional methods are to design numerous controllers and switch among them when needed, or to use adaptive approaches. To this end, research effort has been dedicated to developing flexible methods for updating the model parameters by, for instance, using system identification methods or parameter estimation via neural networks (Källström and Åström, 1981; Kallstrom, 1982; Fossen et al., 1996; Sutton et al., 1997; Mišković et al., 2011; Dai et al., 2012; Wang et al., 2017). In the majority of the aforementioned works, model-based approaches exploiting human knowledge on hydrodynamics and the laws of motion were considered.

*Reinforcement learning (RL)*, also known as neuro-dynamic programming or approximate dynamic programming, is a field of research developed by the Artificial Intelligence (AI) community for achieving optimal sequential decision making under system and environment uncertainty. The roots of RL can be traced back to the 60's and a thorough overview of its evolution can be found in Sutton and Barto (2018) and Bertsekas (2019). Contrary to optimal control theory, RL is based on *evaluative*, rather than instructive, feedback and comes in different forms, which may or may not include partial knowledge of the environment or the system. The process typically involves hand-engineering a reward function, which assigns a reward, or penalty, to the actions that induce desired, or undesired, outcomes, respectively. An RL algorithm is then assigned to find a policy (or controller, in control engineering terminology) that solves the control objective optimally, given the problem constraints and uncertainties. To sum up, RL algorithms use the reward function as a guide, and through trial and error, learn to model the system and its environment, which then leads to a policy that provides an optimal solution to the assigned problem.

Despite a number of successes for RL on simple problems, including algorithms, such as *Q-learning* and *REINFORCE*, the field has seen limited interest. In recent years there has however been a resurgence of interest due to the development of Deep Reinforcement Learning (DRL), starting with Deep Mind developing the *Deep Q-Network* (DQN) algorithm that achieved superhuman performance in several Atari games (Mnih et al., 2013), followed by Deep Mind's *AlphaGo* algorithm becoming the first computer program to beat a human champion in the game of *Go* (Silver et al., 2016). Since then, DRL has been successful in surpassing all previous computer programs in chess and learning how to accomplish complex robotic tasks (Silver et al., 2017; Andrychowicz et al., 2018). Given DRL's ability to tackle problems with high uncertainty, implementations to motion control scenarios involving marine vessels have been presented recently (Shen and Guo, 2016; Zhang et al., 2016; Pham Tuyen et al., 2017; Yu et al., 2017; Cheng and Zhang, 2018; Martinsen and Lekkas, 2018a,b). In most of these works the authors implemented algorithms pertaining to the class of *actor-critic* RL methods, which involves two parts (Konda and Tsitsiklis, 2000): The *actor*, where the gradient of the performance is estimated and the policy parameters are directly updated in a direction of improvement. The main drawbacks of the actor are that it is prone to variance and the new gradient is estimated independently of past estimates. The *critic*, learns an approximation of the value function, leading to an approximate solution to the Bellman or Hamilton-Jacobi-Bellman equation, which then is expected to prescribe a near-optimal policy. The critic's main drawback is that it lacks reliable guarantees in terms of near-optimality of the resulting policy. The actor-critic approach involves the actor improving the policy parameters' estimation based on the approximations learned by the critic. In the case of DRL, one main novelty was the use of two DNNs as function approximators of the policy and the value function, which resulted in considerably improved performance compared to previous approaches. However, DNNs have drawbacks, with some of the most important being lack of transparency and interpretability, lack of robustness, and inability to generalize to situations beyond their past experiences.

In this paper, we follow and extend the work by Kamalapurkar et al. (2018) and Walters et al. (2018) in order to build a trajectory tracking control system for a fully-actuated unmanned surface vehicle (USV). Conceptually, the approach is quite similar to dynamic positioning (DP) (Sørensen, 2011), but extends to higher velocity operational domains, while also trying to optimize tracking performance and compensate for environmental forces (Lekkas and Fossen, 2014). The method combines elements from reinforcement learning, Lyapunov stability theory and system identification: We assume the structure of the vessel model is known but all of its parameters are unknown and have to be estimated online, as well as updated accordingly when the operational conditions change. Then we derive the tracking error dynamics for a generic reference trajectory and a stabilizing parametric control law (the *actor*), whose parameters are estimated during operation.

In order to validate the control scheme, the proposed method was tested in both in simulations, and on a physical model of DNV GL's *ReVolt* platform.

## 2. Reinforcement Learning-Based Trajectory Tracking

In this section we will derive a trajectory tracking control system for fully-actuated USVs. Since the approach is a model based reinforcement learning approach, we will start by looking at how ASVs can be modeled, and how the models can be approximated online using system identification. We will derive a feedforward control law for tracking the desired trajectory, and a feedback control law based on reinforcement learning, for controlling the drift of the vessel in a way that minimizes a given cost function.

### 2.1. Vessel Model

**Figure 1:**
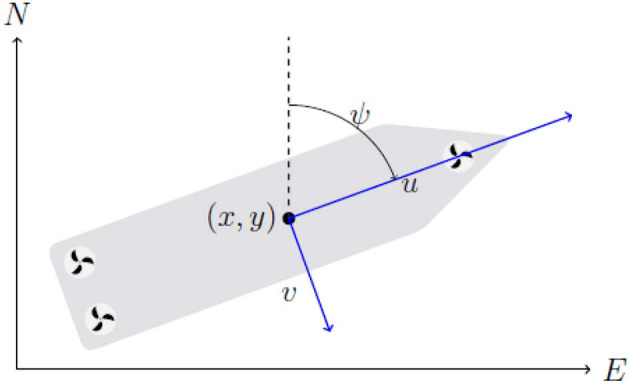
3-DOF vessel centered at (*x, y*), with surge velocity *u*, sway velocity *v*, heading ψ in a North-East-Down (NED) reference frame.

The mathematical model used to describe the system can then be kept reasonably simple by limiting it to the planar position and orientation of the vessel. The motion of a surface vessel can be represented by the pose vector **η** = [*x, y*, ψ]^⊤^ ∈ ℝ^2^ × 𝕊, and velocity vector **ν** = [*u, v, r*]^⊤^ ∈ ℝ^3^. Here, (*x, y*) describe the Cartesian position in the earth-fixed reference frame, ψ is yaw angle, (*u, v*) is the body fixed linear velocities, and *r* is the yaw rate, an illustration is given in Figure 1. Using the notation in Fossen (2011) we can describe a 3-DOF vessel model as follows

(1) $$\begin{array}{l} \;\overset{∙}{\eta }=J\left(\eta \right)\nu ,\\ M\overset{∙}{\nu }+D\left(\nu \right)\nu +C\left(\nu \right)\nu =\tau_{\text{Thrust}}+\tau_{\text{Environment}} \end{array}$$

where ***M*** ∈ ℝ^3 × 3^, ***D***(**ν**) ∈ ℝ^3 × 3^, ***C***(**ν**) ∈ ℝ^3 × 3^, $\tau_{\text{Thrust}},\tau_{\text{Environment}}\in {\mathbb{R}}^{3}$ and ***J***(**η**) ∈ *SO*(3) are the inertia matrix, damping matrix, coriolis matrix, control input vector, environmental forces, and rotation matrix, respectively. The rotational matrix ***J***(**η**) ∈ *SO*(3) is given by

(2) $$\begin{array}{r} J\left(\eta \right)=\left[\begin{array}{ccc} \cos\left(\psi \right) & -\sin\left(\psi \right) & 0\\ \sin\left(\psi \right) & \cos\left(\psi \right) & 0\\ 0 & 0 & 1 \end{array}\right] \end{array}$$

and is the rotation from the body frame to the earth-fixed North East Down (NED) reference frame.

### 2.2. Model Approximation

While the structure of a vessel model, as given above, is well-known, the model parameters are often difficult to find. For our approach we wish to make as few assumptions on the parameters of the vessel model as possible, and use online system identification in order to model the vessel based on gathered data. For this we assume that we know the model structure as given in (1), but that the model parameters are unknown. Splitting the model into a known and unknown part, we get the following:

(3) $$\begin{array}{r} \overset{∙}{x}=f_{\theta }\left(x\right)+f_{1}\left(x\right)+g\left(x\right)u \end{array}$$

where *f*_1_(***x***) and *g*(***x***) are known, and *f*_**θ**_(***x***) is unknown. For the vessel model in (1), with the state vector ***x*** = [**η**, **ν**]^⊤^ and the control vector ***u*** = **τ**_Thrust_. We have the following:

$$\begin{array}{l} f_{\theta }\left(x\right)=\left[\begin{array}{c} 0_{3\times 1}\\ -M^{-1}\left(D\left(\nu \right)\nu +C\left(\nu \right)\nu -\tau_{\text{Environment}}\right) \end{array}\right]\\ f_{1}\left(x\right)=\left[\begin{array}{c} J\left(\eta \right)\nu \\ 0_{3\times 1} \end{array}\right]\\ g\left(x\right)=\left[\begin{array}{c} 0_{3\times 3}\\ M^{-1} \end{array}\right] \end{array}$$

hence we assume the mass matrix is known, but the damping and coriolis matrix are unknown. For the damping and coriolis matrices we assume the vessel has port starboard symmetry, from Fossen (2011) this gives the following structure.

(4) $$\begin{array}{r} C\left(\nu \right)=\left[\begin{array}{ccc} 0 & 0 & Y_{\overset{∙}{v}}\cdot v+Y_{\overset{∙}{r}}\cdot r\\ 0 & 0 & -X_{\overset{∙}{u}}\cdot u\\ -Y_{\overset{∙}{v}}\cdot v+Y_{\overset{∙}{r}}\cdot r & X_{\overset{∙}{u}}\cdot u & 0 \end{array}\right] \end{array}$$

(5) $$\begin{array}{r} D\left(\nu \right)=\left[\begin{array}{ccc} -X_{u}-X_{|u|u}\cdot |u| & 0 & 0\\ 0 & -Y_{v}-Y_{|v|v}\cdot |v|-Y_{|r|v}\cdot |r| & -Y_{r}-Y_{|v|r}\cdot |v|-Y_{|r|r}\cdot |r|\\ 0 & -N_{v}-N_{|v|v}\cdot |v|-N_{|r|v}\cdot |r| & -N_{r}-N_{|v|r}\cdot |v|-N_{|r|r}\cdot |r| \end{array}\right] \end{array}$$

For the damping matrix ***D***(**ν**), both linear and non-linear terms are included. The linear terms are important for low speed maneuvering and station keeping, while ensuring the velocity converges exponentially to zero. The non-linear terms are required as they dominate at higher velocities. This ensures that the model is able to handle a large range of velocities, i.e., it can be used for both high speed trajectory tracking and low speed station keeping and dynamic positioning. For the coriolis matrix, we use only the added mass terms. Since the structure of the rigid body, and added mass is the same for the coriolis matrix, the coriolis matrix given above will be able to capture both the added mass and rigid body dynamics.

In addition to learning the vessel dynamics, we also wanted to be able to compensate for environmental forces. In order to allow for the environmental forces to be learned, they are modeled as an additional unknown pressure vector $p_{\text{env}}^{\text{NED}}={\left[p_{\text{North}},p_{\text{East}},0\right]}^{⊤}$ assumed constant in the NED frame. The resulting force in the body frame is then assumed to be proportional to the cross sectional area of the vessel times the pressure in the body frame, giving the following relationship.

(6) $$\begin{array}{l} \tau_{\text{Environment}}^{\text{body}}=\text{diag}\left(\left[w,l,0\right]\right)J^{⊤}\left(\nu \right)p_{\text{Environment}}^{\text{NED}} \end{array}$$

where *w* and *l* are the width and length of the vessel, respectively, note that for better accuracy calculated pressure coefficients based on the design of the hull may be used instead of the width and length. The unknown parameters are

(7) $$\begin{array}{l} \theta =[X_{\dot{u}},Y_{\dot{v}},Y_{\dot{r}},X_{u},Y_{v},Y_{r},N_{v},N_{r},X_{|u|u},Y_{|v|v},Y_{|v|r},Y_{|r|v},Y_{|r|r},\\ \;N_{|v|v},N_{|v|r},N_{|r|v},N_{|r|r},p_{\text{North}},p_{\text{East}}{]}^{⊤} \end{array}$$

and the function *f*_**θ**_(***x***) can be written as a linear function in **θ**:

(8) $$\begin{array}{r} f_{\theta }\left(x\right)=Y\left(x\right)\theta \end{array}$$

where *Y*(***x***) is:

(9) $$\begin{array}{l} Y\left(x\right)=\left[\begin{array}{c} 0_{3\times 3}\\ -M^{-1} \end{array}\right]\;\left[\begin{array}{ccccccccccccccccccc} 0 & v\cdot r & r^{2} & -u & 0 & 0 & 0 & 0 & -|u|u & 0 & 0 & 0 & 0 & 0 & 0 & 0 & 0 & w\cos\psi & w\sin\psi \\ -u\cdot r & 0 & 0 & 0 & -v & -r & 0 & 0 & 0 & -|v|v & -|v|r & -|r|v & -|r|r & 0 & 0 & 0 & 0 & -l\sin\psi & l\cos\psi \\ u\cdot v & -v\cdot u & -r\cdot u & 0 & 0 & 0 & -v & -r & 0 & 0 & 0 & 0 & 0 & -|v|v & -|v|r & -|r|v & -|r|r & 0 & 0 \end{array}\right] \end{array}$$

We therefore obtain the following parametric model:

(10) $$\begin{array}{r} \overset{∙}{x}=Y\left(x\right)\theta +f_{1}\left(x\right)+g\left(x\right)u, \end{array}$$

which is linear in the parameters **θ**.

#### 2.2.1. Model Assumptions

- The vessel is port starboard symmetric, with a structure as given as in (Figure 1).
- The vessel dampening is linear and quadratic with respect to the linear and angular velocity.
- Environmental forces are constant in the NED frame, and proportional to vessel cross section.
- The vessel is fully actuated.

### 2.3. Trajectory Tracking

In this section we will develop an adaptive feedforward control law which given a time-varying trajectory, finds the control inputs required to follow the trajectory, given the model approximation found in the previous section.

When the control objective is to track a bounded continuously differentiable signal ***x***_*d*_, the dynamics of the tracking error ***e*** = ***x*** − ***x***_*d*_ can be written as

(11) $$\begin{array}{r} \overset{∙}{e}=f\left(x\right)+g\left(x\right)u-{\overset{∙}{x}}_{d} \end{array}$$

Assuming *g*(***x***) is bounded and has full column rank for all ***x*** (Kamalapurkar et al., 2018), then the system is controllable, which in this case holds as the vessel is fully actuated. This gives the feedforward control for the reference trajectory as :

(12) $$\begin{array}{r} u_{d}\left(x_{d},{\overset{∙}{x}}_{d}\right)=g^{+}\left(x_{d}\right)\left({\overset{∙}{x}}_{d}-f\left(x_{d}\right)\right) \end{array}$$

where *g*^+^ is the left Moore–Penrose pseudo-inverse, given as *g*^+^ = (^*g*^⊤^*g*)−1^*g*^⊤^. Using a reference model ${\overset{∙}{x}}_{d}=h_{d}\left(x_{d}\right)$, the feedforward control for the reference trajectory can be written as:

(13) $$\begin{array}{r} u_{d}\left(x_{d}\right)=g^{+}\left(x_{d}\right)\left(h_{d}\left(x_{d}\right)-f\left(x_{d}\right)\right) \end{array}$$

We can then formulate the tracking problem as the following time-invariant optimal control problem.

(14) $$\begin{array}{r} \underset{\overset{∙}{\zeta }}{\underset{︸}{\left[\begin{array}{c} \overset{∙}{e}\\ {\overset{∙}{x}}_{d} \end{array}\right]}}=\underset{F\left(\zeta \right)}{\underset{︸}{\left[\begin{array}{c} f\left(e+x_{d}\right)+g\left(e+x_{d}\right)u_{d}\left(x_{d}\right)\\ h_{d}\left(x_{d}\right) \end{array}\right]}}+\underset{G\left(\zeta \right)}{\underset{︸}{\left[\begin{array}{c} g\left(e+x_{d}\right)\\ 0 \end{array}\right]}}\pi \end{array}$$

Where **π** is an input correction for the drift dynamics, which we will define in the next section. Given the parametric model in (10), the parametric version of the tracking problem is given as:

(15) $$\begin{array}{l} \underset{\overset{∙}{\zeta }}{\underset{︸}{\left[\begin{array}{c} \overset{∙}{e}\\ {\overset{∙}{x}}_{d} \end{array}\right]}}=\underset{F\left(\zeta ;\theta \right)}{\underset{︸}{\left[\begin{array}{c} Y\left(e+x_{d}\right)\theta +f_{1}\left(e+x_{d}\right)+g\left(e+x_{d}\right)u_{d}\left(x_{d};\theta \right)\\ h_{d}\left(x_{d}\right) \end{array}\right]}}\\ \;+\underset{G\left(\zeta \right)}{\underset{︸}{\left[\begin{array}{c} g\left(e+x_{d}\right)\\ 0 \end{array}\right]}}\pi \end{array}$$

where the parametric feedforward control for the reference trajectory ***u***_*d*_(***x***_*d*_; **θ**) is given as:

(16) $$\begin{array}{r} u_{d}\left(x_{d};\theta \right)=g^{+}\left(x_{d}\right)\left(h_{d}\left(x_{d}\right)-Y\left(x_{d}\right)\theta -f_{1}\left(x_{d}\right)\right) \end{array}$$

Given the formulation above, with the feedforward control for the reference trajectory ***u***_*d*_(***x***_*d*_), and the optimal model parameters θ^*^, the exact feedforward control for the reference trajectory is possible to compute. The dynamics above guarantee trajectory tracking when $\overset{∙}{e}=0$, i.e., when the tracking error is zero. When the tracking error is not zero however, we need to control the drift dynamics in order to ensure convergence to the desired trajectory by designing the feedback control **π**(*t*) such that $\underset{t\to \infty }{\lim}e\left(t\right)=0$. The objective of the optimal control problem is to design the feedback control law **π**(*t*) such that it minimizes a given cost function.

### 2.4. Approximate Optimal Control of Drift Dynamics

In the previous section we developed a feedforward control law ***u***_*d*_(***x***_*d*_; **θ**) for tracking a desired trajectory. Due to inaccuracies in model approximation and disturbances, using only the feedforward control law, the vessel will experience drift. In order to compensate for the inevitable drift, we will in this section develop a feedback control law **π**(·), which controls the drift dynamics in a way that optimizes a given cost function. We will additionally show how the parameters of the feedback control law can be learned by using reinforcement learning.

The optimal control problem we wish to solve is that of minimizing the cost function:

(17) $$\begin{array}{r} J\left(\zeta ,\pi \right)=\int_{t_{0}}^{\infty }r\left(\zeta \left(\tau \right),\pi \left(\tau \right)\right)d\tau \end{array}$$

Where *r*(·) is scalar function defining the local cost, and should not be confused with the yaw rate. The cost function is defined as:

(18) $$\begin{array}{r} r\left(\zeta ,\pi \right)=Q\left(\zeta \right)+\pi^{⊤}R\pi \end{array}$$

where ***R*** ≻ 0 is a positive definite symmetric matrix. And *Q*(**ζ**) is a positive definite function. Assuming that a minimizing control policy **π**(·) exists, the optimal value function is given as:

(19) $$\begin{array}{r} V^{*}(\zeta )=\underset{{\;}_{\pi (\tau ),\;\tau \in [t_{0},\infty )}}{\min}\int_{t_{0}}^{\infty }r(\zeta (\tau ),\pi (\tau ))d\tau \end{array}$$

We can now note that for a small time step Δ*t*, the above expression can be formulated as:

$$\begin{array}{l} V^{*}(\zeta (t))=\underset{{\;}_{{\;}_{\pi (\tau ),\;\tau \in [t,t+\Delta t)}}}{\min}\int_{t}^{t+\Delta t}r(\zeta (\tau ),\pi (\tau ))d\tau +V^{*}(\zeta (t+\Delta t)) \end{array}$$

Taking the limit of this as Δ*t* → 0, for the optimal value function under the optimal policy, we get (Doya, 2000):

$$\begin{array}{l} V^{*}\left(\zeta \left(t\right)\right)=\underset{\pi \left(t\right)}{\min}r\left(\zeta \left(t\right),\pi \left(t\right)\right)+V^{*}\left(\zeta \left(t\right)\right)+{\dot{V}}^{*}\left(\zeta \left(t\right)\right) \end{array}$$

Simplifying this we get the Hamilton-Jacobi-Bellman (HJB) equation for the optimal control problem as follows:

(20) $$\begin{array}{l} H^{*}={\overset{∙}{V}}^{*}\left(\zeta \right)+r\left(\zeta ,\pi^{*}\left(\zeta \right)\right)\\ \;=\nabla_{\zeta }V^{*}{\left(\zeta \right)}^{⊤}\overset{∙}{\zeta }+r\left(\zeta ,\pi^{*}\left(\zeta \right)\right)\\ \;=\nabla_{\zeta }V^{*}{\left(\zeta \right)}^{⊤}\left(F\left(\zeta \right)+G\left(\zeta \right)\pi^{*}\left(\zeta \right)\right)+r\left(\zeta ,\pi^{*}\left(\zeta \right)\right)=0 \end{array}$$

Where H^*^, π^*^ and V^*^ is the optimal hamiltonian, policy and value function, respectively. From calculus of variation (Liberzon, 2011) we have the Hamiltonian minimization condition, which states that a value function V is the optimal Value function if and only if there exists a controller **π**(·) and trajectory **ζ**(·) under **π**(·) satisfy the equation:

(21) $$\begin{array}{c} \nabla_{\zeta }V{\left(\zeta \right)}^{⊤}\left(F\left(\zeta \right)+G\left(\zeta \right)\pi \left(\zeta \right)\right)+r\left(\zeta ,\pi \left(\zeta \right)\right)\\ =\underset{\hat{\pi }\in U}{\min}\left\{\nabla_{\zeta }V{\left(\zeta \right)}^{⊤}\left(F\left(\zeta \right)+G\left(\zeta \right)\hat{\pi }\left(\zeta \right)\right)+r\left(\zeta ,\hat{\pi }\left(\zeta \right)\right)\right\} \end{array}$$

The necessary conditions for this to hold are:

(22) $$\begin{array}{l} \nabla_{\pi }\left(\nabla_{\zeta }V{\left(\zeta \right)}^{⊤}\left(F\left(\zeta \right)+G\left(\zeta \right)\pi \left(\zeta \right)\right)+r\left(\zeta ,\pi \left(\zeta \right)\right)\right)=0 \end{array}$$

which gives the closed form solution of the optimal controller as:

$$\begin{array}{l} \begin{array}{c} G^{⊤}\left(\zeta \right)\left(\nabla_{\zeta }V\left(\zeta \right)\right)+\nabla_{\pi }r\left(\zeta ,\pi \right)=0\Leftrightarrow \\ \;2R\pi =-G^{⊤}\left(\zeta \right)\left(\nabla_{\zeta }V\left(\zeta \right)\right)\Leftrightarrow \end{array} \end{array}$$

(23) $$\begin{array}{l} \pi^{*}\left(\zeta \right)=-\frac{1}{2}R^{-1}G^{⊤}\left(\zeta \right)\left(\nabla_{\zeta }V\left(\zeta \right)\right) \end{array}$$

Hence assuming that an optimal controller exists, the closed form solution given by the HJB equation is given by (23). Note that the value function is assumed time independent, and hence we are looking for a stationary solution of the HJB equation. This holds true, as the reformulation into a trajectory tracking problem (15) gives a time independent system.

The Universal Approximation theorem (Kamalapurkar et al., 2018, Property 2.3) states that a single layer neural network can simultaneously approximate a function and its derivative given a sufficiently large number of basis functions. Using this, we can approximate any continuous function as:

(24) $$\begin{array}{r} V\left(x\right)=W^{⊤}\sigma \left(x\right)+\epsilon \left(x\right) \end{array}$$

where ***W*** is the weighting matrix, σ(***x***) is the vector of basis functions, and ϵ(***x***) is the approximation error, which can be made arbitrarily small by increasing the number of basis functions. Note that the basis functions can here be chosen to be any parameterization, such as Radial-Basis functions, polynomials or even a Fourier series. Using this we can represent the value function as a neural network which is linear in the parameters, giving the optimal value function:

(25) $$\begin{array}{r} V^{*}\left(\zeta \right)=W^{⊤}\sigma \left(\zeta \right)+\epsilon \left(\zeta \right) \end{array}$$

and the optimal policy as a feedback control law on the form:

(26) $$\begin{array}{l} \pi^{*}\left(\zeta \right)=-\frac{1}{2}R^{-1}G^{⊤}\left(\zeta \right)\left(\nabla_{\zeta }\sigma {\left(\zeta \right)}^{⊤}W+\nabla_{\zeta }\epsilon^{⊤}\left(\zeta \right)\right) \end{array}$$

By making the parameterizations sufficiently rich, we make the approximation error small. We can then use the approximations given below, for the value function and control policy, respectively.

(27) $$\begin{array}{r} \hat{V}\left(\zeta ;{\hat{W}}_{c}\right)={\hat{W}}_{c}^{⊤}\sigma \left(\zeta \right) \end{array}$$

(28) $$\begin{array}{r} \hat{\pi }\left(\zeta ;{\hat{W}}_{a}\right)=-\frac{1}{2}R^{-1}G^{⊤}\left(\zeta \right)\nabla_{\zeta }\sigma {\left(\zeta \right)}^{⊤}{\hat{W}}_{a} \end{array}$$

In order to find the parameters ${\hat{W}}_{c}$ and ${\hat{W}}_{a}$, we will in the next section find update laws, based on reinforcement learning, to be able to optimize performance online.

**Figure 2:**
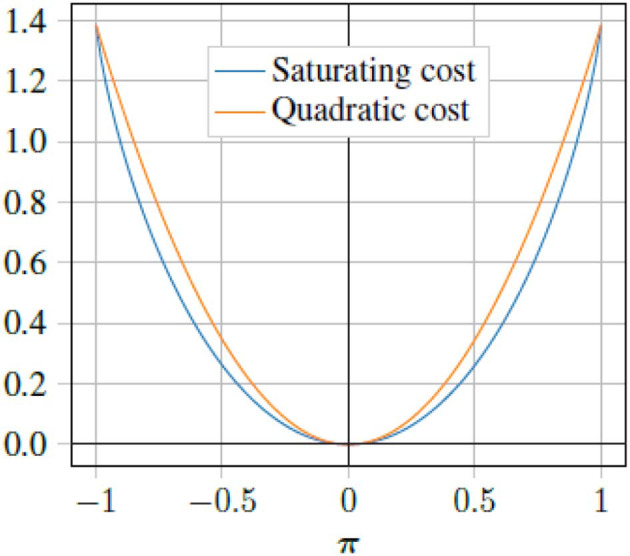
Comparison between saturating cost (29) and quadratic cost (18). Here the quadratic cost is scaled by ln(4).

Unfortunately, policy (28) does not account for the saturating constraints, such as the maximum force the actuators of the physical vessel can produce. In order to account for the actuator limitations, we propose a different control policy which uses a saturating function (Doya, 2000) in order to avoid this problem. Using the following cost function:

(29) $$\begin{array}{r} r\left(\zeta ,\pi \right)=Q\left(\zeta \right)+2\overset{m}{\underset{i=1}{\sum }}r_{i}\int_{0}^{\pi_{i}}\tanh^{-1}\left(\xi \right)d\xi \end{array}$$

where *r*_*i*_ is the *i*th entry of the diagonal of ***R***, i.e., ***R*** = diag([*r*_1_, *r*_2_…*r*_*m*_]). Figure 2 shows a comparison of the saturating input cost, and a pure quadratic cost. Performing the same analysis as for the quadratic penalty, we can get the following saturating control law:

(30) $$\begin{array}{l} \pi^{*}\left(\zeta \right)=-\tanh\left(\frac{1}{2}R^{-1}G^{⊤}\left(\zeta \right)\left(\nabla_{\zeta }V\left(\zeta \right)\right)\right) \end{array}$$

Since tanh(·) saturates at ±1, this means that the feedback control law **π** will saturate at ±1, the outputs can then be easily scaled to fit other bounds. It can be shown that since tanh(·) is a monotonically increasing continuously differentiable function, the control law satisfies the first order necessary conditions, and the second order sufficient conditions of the Hamiltonian minimization condition. This means that if an optimal controller exists the closed form solution is given by (30). Using an approximation we get the following approximate optimal policy

(31) $$\begin{array}{l} \hat{\pi }\left(\zeta ;{\hat{W}}_{a}\right)=-\tanh\left(\frac{1}{2}R^{-1}G^{⊤}\left(\zeta \right)\nabla_{\zeta }\sigma {\left(\zeta \right)}^{⊤}{\hat{W}}_{a}\right) \end{array}$$

It should be noted, that while the policy in (31) uses a value function approximation in order to approximate the optimal policy, the parameters ${\hat{W}}_{a}$ are not the same as the parameters ${\hat{W}}_{c}$ in the value function approximation in (27). In this way we can separate the learning of the policy and value function, this is known as an actor critic method, where the value function is known as the critic, and the policy is known as an actor. The intuitive reason for doing this, is that it allows the critic to learn the value function resulting from the behavior of the policy, and in this way it ca critique the policy. Similarly, the policy or actor, can learn to improve its performance based on the criticism of the critic. How the learning is performed is further discussed in the next section.

### 2.5. Update Laws

Now that we have expressed the control laws $u_{d}\left(x_{d};\hat{\theta }\right)$, $\hat{\pi }\left(\zeta ;{\hat{W}}_{a}\right)$ and value function $\hat{V}\left(\zeta ;{\hat{W}}_{c}\right)$, the challenge becomes finding update laws for the parameters of the system identification $\hat{\theta }$, the critic ${\hat{W}}_{c}$ and the actor ${\hat{W}}_{a}$. For the model parameters $\hat{\theta }$, we will use methods from system identification and adaptive control, to try to optimize the fit between the parameterized model, and the observed vessel states. For the actor and critic parameters ${\hat{W}}_{a}$ and ${\hat{W}}_{c}$, we will use model based reinforcement learning to find the parameters that gives the optimal value function, and consequently the optimal feedback control policy.

For the system identification parameters $\hat{\theta }$, the goal is to find the parameters for which the model behaves as similarly as possible to the observed behavior. Running our physical system, and collecting observations $\left({\overset{∙}{x}}_{i},x_{i},u_{i}\right)\;i\in 1,2,\ldots ,N$, we can formulate a least squares optimization problem for finding the parameters that minimize the difference between the observed state derivative ${\overset{∙}{x}}_{i}$ and the parametric model (3) as follows.

$$\begin{array}{l} \theta^{*}=\text{arg}\underset{\hat{\theta }}{\min}\underset{L\left(\hat{\theta }\right)}{\underset{︸}{\overset{N}{\underset{i=1}{\sum }}\frac{1}{2}||{\overset{∙}{x}}_{i}-Y\left(x_{i}\right)\hat{\theta }-f_{1}\left(x_{i}\right)-g\left(x_{i}\right)u_{i}{||}_{2}^{2}}} \end{array}$$

This is a linear least squares optimization problem for which there exists a closed form solution, however we can also solve the problem by performing stochastic gradient decent on the parameters $\hat{\theta }$, as follows:

$$\begin{array}{l} \hat{\theta }\leftarrow \hat{\theta }-\nabla_{\hat{\theta }}L\left(\hat{\theta }\right) \end{array}$$

The gradient decent law above, works in discrete iteration, however we can reformulate it as a an ordinary differential equation (ODE). Doing some further changes motivated by the stability analysis of the convergence of the parameter estimates, we get the concurrent learning based approach proposed in Chowdhary and Johnson (2011b) as:

(32) $$\begin{array}{l} \dot{\hat{\theta }}(t)=\Gamma_{\theta }Y^{⊤}(x(t))\overset{\sim }{x}(t)+\frac{k_{\theta }}{N}\Gamma_{\theta }\sum_{i=1}^{N}Y^{⊤}(x_{i})({\overset{˙}{x}}_{i}-f_{1}(x_{i})\\ \;-g(x_{i})u_{i}-Y(x_{i})\hat{\theta }) \end{array}$$

where **Γ**_θ_ is a parameter weight matrix, and *k*_θ_ is a scalar weight factor. Assuming that the prerecorded data is sufficiently rich such that the matrix $\sum_{i=1}^{N}Y^{⊤}\left(x_{i}\right)Y\left(x_{i}\right)$ is full rank, the parameter error can be shown to converge. As the convergence rate of the system identifier is proportional to the minimum singular value of $\sum_{i=1}^{N}Y^{⊤}\left(x_{i}\right)Y\left(x_{i}\right)$, replacing data in the data stack can be done by using a singular value maximizing algorithm (Chowdhary and Johnson, 2011a) in order to get faster convergence. Note, that since we are assuming a sufficiently rich prerecorded data set, we no longer need persistence of excitation (PE), in order to guarantee parameter convergence.

In order to find the update laws for the critic or value function parameters ${\hat{W}}_{c}$, we need a way of evaluating the optimality of the value function given the current parameters. For this we look back at the HJB Equation (20) given as:

$$\begin{array}{l} 0=r\left(\zeta ,\pi^{*}\left(\zeta \right)\right)+\nabla_{\zeta }V^{*}{\left(\zeta \right)}^{⊤}\left(F\left(\zeta \right)+G\left(\zeta \right)\pi^{*}\left(\zeta \right)\right) \end{array}$$

Substituting the estimates $\hat{V}$ and $\hat{\pi }$ for the optimal value function V^*^ and optimal policy **π**, we can formulate the Bellman error as the error in the HJB equation as follows:

(33) $$\begin{array}{l} \delta \left(\zeta ;\hat{\theta },{\hat{W}}_{c},{\hat{W}}_{a}\right)=\underset{r\left(\zeta ,\hat{\pi }\left(\zeta ;{\hat{W}}_{a}\right)\right)}{\underset{︸}{Q\left(\zeta \right)+{\hat{\pi }}^{⊤}\left(\zeta ;{\hat{W}}_{a}\right)R\hat{\pi }\left(\zeta ;{\hat{W}}_{a}\right)}}\\ \;+\nabla_{\zeta }\hat{V}{\left(\zeta ;{\hat{W}}_{c}\right)}^{⊤}\left(F\left(\zeta ;\hat{\theta }\right)+G\left(\zeta \right)\hat{\pi }\left(\zeta ;{\hat{W}}_{a}\right)\right) \end{array}$$

The Bellman error can intuitively be thought of as the error between the optimal value function under the policy, and the estimates. Since the goal for the value function or critic is to find the parameters ***W***_*c*_ that best approximates the value function, a natural choice becomes to find the parameters that minimize the bellman error. With reinforcement learning we can use a data stack of prerecorded state transitions $\zeta_{i}\left(t\right)={\left[x_{i}-x_{d,i},x_{d,i}\right]}^{⊤}\;i\in 1,2,\ldots N$, to formulate the following optimization problem:

$$\begin{array}{l} \underset{{\hat{W}}_{c}}{\min}\overset{N}{\underset{i=1}{\sum }}\frac{1}{2}\delta {\left(\zeta_{i};\hat{\theta },{\hat{W}}_{c},{\hat{W}}_{a}\right)}^{2} \end{array}$$

This is a non-linear optimization problem, but we may again use a methods like gradient decent in order to iteratively learn parameters that improve the optimization problem given above. Writing the gradient decent in terms of an ODE, and making some changes motivated by a stability analysis (Kamalapurkar et al., 2018). A least-squares update law with forgetting factor (Ioannou and Sun, 2012) can be formulated for the critic as follows:

(34) $$\begin{array}{l} {\overset{∙}{\hat{W}}}_{c}\left(t\right)=-k_{c,1}\Gamma \left(t\right)\frac{\omega \left(\zeta \left(t\right),t\right)}{\rho \left(\zeta \left(t\right),t\right)}\hat{\delta }\left(\zeta \left(t\right),t\right)-\frac{k_{c,2}}{N}\Gamma \left(t\right)\\ \;\overset{N}{\underset{i=1}{\sum }}\frac{\omega \left(\zeta_{i}\left(t\right),t\right)}{\rho_{i}\left(\zeta_{i}\left(t\right),t\right)}\hat{\delta }\left(\zeta_{i}\left(t\right),t\right) \end{array}$$

(35) $$\begin{array}{l} \overset{∙}{\Gamma }\left(t\right)=\{\begin{array}{ll} \beta \Gamma \left(t\right)-k_{c,1}\Gamma \left(t\right)\frac{\omega \left(\zeta \left(t\right),t\right)\omega^{⊤}\left(\zeta \left(t\right),t\right)}{\rho^{2}\left(\zeta \left(t\right),t\right)}\Gamma \left(t\right) & \text{If}||\Gamma ||\leq \bar{\Gamma }\\ 0 & \text{Otherwise} \end{array} \end{array}$$

In critic update law above *k*_*c*, 1_ and *k*_*c*, 2_ are scalar learning rates, while **Γ** is an adaptive weight matrix, and β is a scalar forgetting factor, which controls how previous data samples are discounted. For brevity of notation we used the functions **ω**(·), ρ(·), and $\hat{\delta }\left(\cdot \right)$ defined as:

$$\begin{array}{l} \omega \left(\zeta ,t\right)=\nabla_{\zeta }\sigma \left(\zeta \right)\left(F\left(\zeta ;\hat{\theta }\left(t\right)\right)+G\left(\zeta \right)\hat{\pi }\left(\zeta ;{\hat{W}}_{a}\left(t\right)\right)\right)\\ \rho \left(\zeta ,t\right)=1+\omega^{⊤}\left(\zeta ,t\right)\Gamma \left(t\right)\omega \left(\zeta ,t\right)\\ \hat{\delta }\left(\zeta ,t\right)=\delta \left(\zeta ;\hat{\theta }\left(t\right),{\hat{W}}_{c}\left(t\right),{\hat{W}}_{a}\left(t\right)\right) \end{array}$$

Here, **ω** can be considered a regressor vector, while ρ is a normalization factor, and $\hat{\delta }$ the Bellman error.

The actor update law (36) is chosen such that it learns from the critic, while at the same time trying to stay close to the initial control law.

(36) $$\begin{array}{l} {\overset{∙}{\hat{W}}}_{a}\left(t\right)=\text{proj}\left(-k_{a,1}\left({\hat{W}}_{a}\left(t\right)-{\hat{W}}_{c}\left(t\right)\right)-k_{a,2}\left({\hat{W}}_{a}\left(t\right)-W_{0}\right)\right) \end{array}$$

In the actor update law above, the first term will make the actor parameters follow the critic parameters, while the second term will try to keep the actor parameters close to the initial parameters ***W***_0_. *k*_*a*, 1_ and *k*_*a*, 2_ are scalar learning rates for the two terms. A smooth projection (Ioannou and Sun, 2012) is added such that the actor weights are within a predefined region, for which the control law is stable. Any smooth projection can be chosen, however we chose a projection ensuring the actor weights were bounded within a region of the initial weights ***W***_0_.

### 2.6. Stability Analysis

For the system identification parameters **θ**, we consider the candidate Lyapunov function:

(37) $$\begin{array}{r} V_{p}\left(x\right)={\tilde{\theta }}^{⊤}\Gamma_{\theta }^{-1}\tilde{\theta }, \end{array}$$

where $\tilde{\theta }=\hat{\theta }-\theta^{*}$ is the difference between the predicted and optimal model parameters. Assuming the system is time invariant (including time invariant environmental forces in the NED frame), and given a positive definite weighting matrix **Γ**_**θ**_. The time derivative of the candidate Lyapunov function is:

(38) $$\begin{array}{l} {\overset{∙}{V}}_{P}\left(x\right)=2{\tilde{\theta }}^{⊤}\Gamma_{\theta }^{-1}\overset{∙}{\theta }\\ \;=2{\tilde{\theta }}^{⊤}\Gamma_{\theta }^{-1}\Gamma_{\theta }Y{\left(x\right)}^{⊤}\tilde{x}+\frac{2k_{\theta }}{N}{\tilde{\theta }}^{⊤}\Gamma_{\theta }^{-1}\Gamma_{\theta }\overset{N}{\underset{i=1}{\sum }}Y^{⊤}\left(x_{i}\right){\tilde{x}}_{i}. \end{array}$$

Using the fact that: $\tilde{x}=\overset{∙}{x}-f_{1}\left(x\right)-Y\left(x\right)\hat{\theta }-g\left(x\right)\tau =-Y\left(x\right)\tilde{\theta }$ we get:

(39) $$\begin{array}{l} {\overset{∙}{V}}_{P}\left(x\right)=-2{\tilde{x}}^{⊤}\tilde{x}-\frac{2k_{\theta }}{N}{\tilde{\theta }}^{⊤}\overset{N}{\underset{i=1}{\sum }}\left(Y^{⊤}\left(x_{i}\right)Y\left(x_{i}\right)\right)\tilde{\theta }\leq 0, \end{array}$$

hence the model error $\tilde{x}$ and parameter error $\tilde{\theta }$ converge exponentially to zero as *t* → ∞. We can also note that the rate of the parameter convergence is given by the singular values of $\sum_{i=1}^{N}\left(Y^{⊤}\left(x_{i}\right)Y\left(x_{i}\right)\right)$.

For the RL update laws in (34)–(36), it can be shown that under a number of strict assumptions, a system on the form given in (15), with an unconstrained policy, is uniformly ultimately bounded in terms of the error dynamics ***e***, as well as the weights and parameters ***W***_*a*_, ***W***_*c*_, and **θ**. The stability analysis can be found in Kamalapurkar et al. (2018). For our purposes, we further constrain the parameters ***W***_*a*_ of the feedback control law by projecting them into a region close to a known stable initial parameterization. Closed loop stability is important for assurance of the control system, this is further discussed in section 4.

### 2.7. Reference Model

When generating a reference path, we must ensure that it is sufficiently smooth in order to be able to say something about the convergence to the path. In practice however, we may have a signal which is discrete, defining the desired pose only at certain times. In order to smooth the trajectory we therefor use a reference model, which tracks the discrete reference pose, and generates a continuous reference trajectory pose $\eta_{d}={\left[x_{d},y_{d},\psi_{d}\right]}^{⊤}$ and velocity vector $\nu_{d}={\left[u_{d},v_{d},r_{d}\right]}^{⊤}$. For the pose we can make a reference model on the following form:

(40) $$\begin{array}{l} \left[\begin{array}{c} {\overset{∙}{\eta }}_{d}\\ {\ddot{\eta }}_{d}\\ {\overset{⃛}{\eta }}_{d} \end{array}\right]=\left[\begin{array}{ccc} 0 & I & 0\\ 0 & 0 & I\\ -\Omega^{3} & -\left(2\Delta +I\right)\Omega^{2} & -\left(2\Delta +I\right)\Omega \end{array}\right]\left[\begin{array}{c} \eta_{d}\\ {\overset{∙}{\eta }}_{d}\\ {\ddot{\eta }}_{d} \end{array}\right]\\ \;+\left[\begin{array}{c} 0\\ 0\\ \Omega^{3} \end{array}\right]\eta_{ref} \end{array}$$

Where **Ω** = diag([ω_1_, …, ω_*n*_]) and **Δ** = diag([δ_1_, …, δ_*n*_]). Choosing **Δ** = ***I*** ensures the reference model is critically damped, while **Ω** controls the rate of convergence of the states. We must also generate the velocity vector, however based on the pose, the velocity can be calculated as:

(41) $$\begin{array}{l} \nu_{d}=J^{⊤}\left(\eta_{d}\right){\overset{∙}{\eta }}_{d}\\ {\overset{∙}{\nu }}_{d}=-S\left({\left[0,0,r_{d}\right]}^{⊤}\right)J^{⊤}\left(\eta_{d}\right){\overset{∙}{\eta }}_{d}+J^{⊤}\left(\eta_{d}\right){\ddot{\eta }}_{d} \end{array}$$

where $-S\left({\left[0,0,r_{d}\right]}^{⊤}\right)J^{⊤}\left(\eta_{d}\right)={\overset{∙}{J}}^{⊤}\left(\eta_{d}\right)$, and ***S***(**ω**) is the skew symmetric matrix:

$$\begin{array}{l} S\left({\left[\omega_{1},\omega_{2},\omega_{3}\right]}^{⊤}\right)=\left[\begin{array}{ccc} 0 & -\omega_{3} & \omega_{2}\\ \omega_{3} & 0 & -\omega_{1}\\ -\omega_{2} & \omega_{1} & 0 \end{array}\right] \end{array}$$

The reason we here use a third order filter for the reference model, is to ensure a smooth pose, velocity, and acceleration, even when a step in the reference is observed. This ensures that the feedforward control for the reference trajectory (16) can track the reference.

**Figure 3:**
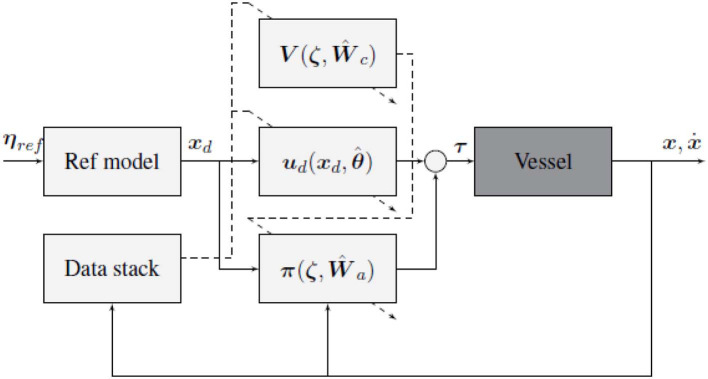
Block diagram of the proposed control scheme, solid lines represent state and control signals, while dashed lines represent adaption signals. Note that the underlying vessel dynamics considered are unknown, and includes thrust allocation and state estimation.

A block diagram of the final control structure is given in Figure 3. The diagram shows how the controller is split into a feedback control law **π**, and a feedforward control law ***u***_*d*_. Where the Reference filter is used to generate the pose and velocity reference ***x***_*d*_, and the data stack collected from observing vessel transitions, is used to update the parameters of the control laws.

## 3. Experiments

**Figure 4:**
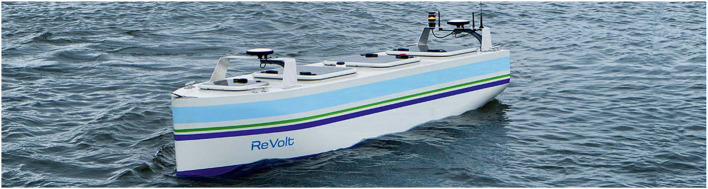
*ReVolt* test platform courtesy of DNV GL.

In this section we present the results form simulations, and sea trials on the *ReVolt* test platform (see Figure 4), when using the control scheme proposed in the previous section. We will first present the implementation details for the for the control algorithm. After that we will briefly present the experimental platform, before finally presenting the simulation, and sea trial results for varying operational conditions. The experiments include both low speed dynamic positioning, and high speed trajectory tracking.

### 3.1. Implementation Details

For the implementation the parameter update laws (32), (35), (35), and (34) were implemented with a 4th order Runge-Kutta integration scheme, with a timestep of 0.1 s. Additionally the reference model in (40) and (41) were implemented, also using a 4th order Runge-Kutta scheme, in order to generate the reference trajectory $x_{d}={\left[\eta_{d},\nu_{d}\right]}^{⊤}$ and its derivative ${\overset{∙}{x}}_{d}=h_{d}={\left[{\overset{∙}{\eta }}_{d},{\overset{∙}{\nu }}_{d}\right]}^{⊤}$.

For the parameterization of the system identifier, the **θ** and *Y*(***x***) were chosen as in (7) and (9), while for the actor and critic, the parameterization σ(**ζ**) was chosen as the vector of all the second order cross terms of the position and velocity error in the body frame $e^{\text{body}}=\left[{\tilde{\eta }}^{\text{body}},\tilde{\nu }\right]$ where ${\tilde{\eta }}^{\text{body}}=J^{⊤}\left(\eta \right)\tilde{\eta }$, giving the following expression:

(42) $$\begin{array}{r} W\sigma \left(\zeta \right)=\underset{x_{i}\in e^{\text{body}}}{\sum }\underset{x_{j}\in e^{\text{body}}}{\sum }w_{i,j}x_{i}x_{j} \end{array}$$

The reason that we use the error in the body frame, is the assumption that the cost is invariant to rotations when in the body frame, as this is the same frame the dynamics of the system are given in. The initial conditions for the actor and critic weights were chosen such that they matched the continuous time algebraic Riccati equation for a simplified linear model of the vessel.

For the control law, the constrained closed form controller (30) was used. And the output was scaled to fit the max thrust and torque $\bar{\tau }=\frac{1}{\sqrt{3}}{\left[50.0,20.0,32.0\right]}^{⊤}$ the vessel is able to produce. While [50.0, 20.0, 32.0]^⊤^ is the max force the vessel is able to produce in each direction individually, due to the coupling between thrusters, we assume the maximum thrust in any given coupled direction can be approximated by the an ellipse with axis lengths 50.0, 20.0, and 32.0. Since the proposed method only allows us to constrain thrust in each individual direction, we use the largest inner approximation of the ellipse as our thrust bound, giving the max thrust and torque as $\bar{\tau }$ given above. It should be noted, that while this constrains the thrust, we can still not guarantee that the vessel is able to produce the desired amount of thrust as $\bar{\tau }$ is only an inner approximation of the elliptic approximation, whereas the true thrust bound may be much more complex. It should also be noted that using the inner approximation $\bar{\tau }$ as a bound, means we are not able to fully utilize the full thrust that the vessel has to offer. One way of solving these issues would be to include the thrust allocation as part of the problem formulation, however this is beyond the scope of this paper. It should also be noted that the desired thrust vector includes both the path tracking control law and drift correction $\tau_{\text{Thrust}}=u_{d}\left(x_{d};\hat{\theta }\right)+\hat{\pi }\left(\zeta ;{\hat{W}}_{a}\right)$ where the saturation is only considered in the drift controller and not the path tracking control law. This means the desired path should be generated in a way that satisfies the thrust constraints.

For the state cost function *Q*(**ζ**) a quadratic cost on the form $Q\left(\zeta \right)={\left[{\tilde{\eta }}^{\text{body}},\tilde{\nu }\right]}^{⊤}Q\left[{\tilde{\eta }}^{\text{body}},\tilde{\nu }\right]$ was chosen, where ***Q*** is a positive definite weight matrix. Given as:

$$\begin{array}{l} Q=\left[\begin{array}{cccccc} 1.0 & 0.0 & 0.0 & 0.0 & 0.0 & 0.0\\ 0.0 & 1.0 & 1.0 & 0.0 & 0.0 & 0.0\\ 0.0 & 1.0 & 10.0 & 0.0 & 0.0 & 0.0\\ 0.0 & 0.0 & 0.0 & 10.0 & 0.0 & 0.0\\ 0.0 & 0.0 & 0.0 & 0.0 & 10.0 & 0.0\\ 0.0 & 0.0 & 0.0 & 0.0 & 0.0 & 10.0 \end{array}\right] \end{array}$$

The weight matrix is given as a mostly diagonal matrix, with a small cross term between the position error in sway direction, and heading error. The cross term is added in order to encourage the vessel to travel in the surge direction when there is a large position error, as this is the most efficient direction of travel.

The data stack that was used consisted of 100 samples, and a singular value maximization scheme was implemented in order to increase the convergence rate. Using a purely singular value maximization based data selection scheme, while giving good performance on a stationary system, does not work for time varying system, and hence does not allow for estimating the slowly varying environmental forces. In order to account for this, weighting of the singular value maximization, and data sample age was used in order to save recent samples with high singular values.

### 3.2. Experimental Platform

The *ReVolt*, shown in Figure 4, is a 1:20 scale model of a autonomous concept vessel developed by DNV GL in collaboration with NTNU. The model is 3 m long, 0.72 m wide, and weighs 257 kg. *ReVolt* has a top speed of 2 knots (~1 m/s) with a total combined engine power of 360 W. The thrust configuration is given as in Figure 1, with two identical stern thrusters, and one slightly less powerful bow thruster, all of which are fully rotatable azimuth thrusters, and are controlled by an optimization based thrust allocation (TA) algorithm. The vessel state is estimated using a non-linear observer consisting of an Extended Kalman Filter (EKF), and combines measurements from a Global Navigation Satellite System (GNSS) with Real-Time Kinematic (RTK) correction data, on board accelerometer, gyroscope, and compass. This provides accurate heading and position down to ±0.2° and ±1 cm. A description of the *ReVolt* hardware and software is given in Table 1.

**Table 1:** *ReVolt* hardware and software specifications.

Onboard computer:	Tank-720
Sensors:	Xsens MTI-G-710 IMU
	Vector VS330 GNSS Receiver
Software:	Linux Ubuntu LTS 16.04
	ROS Kinetic Kame

While the physical vessel was used for the sea trials, a high fidelity Digital Twin of *ReVolt*, developed by DNV GL, was used for simulation. The Digital Twin is based on a full 6DOF model, with parameters identified through tow-tank experiments, as well as frequency domain analysis of a 3D model of the vessel hull. The Digital Twin allowed for rapidly testing how the proposed control scheme performed under ideal conditions, as well as under different sea states, ocean currents and wind conditions.

### 3.3. Simulations and Sea Trials

In order to test the proposed control scheme, a number of experiments were devised. As the control scheme was build to be able to handle both high speed and low speed maneuvering, we wanted to test both, by doing low speed Dynamic Positioning (DP), as well as higher speed path tracking.

#### 3.3.1. Dynamic Positioning (DP)

**Figure 5:**
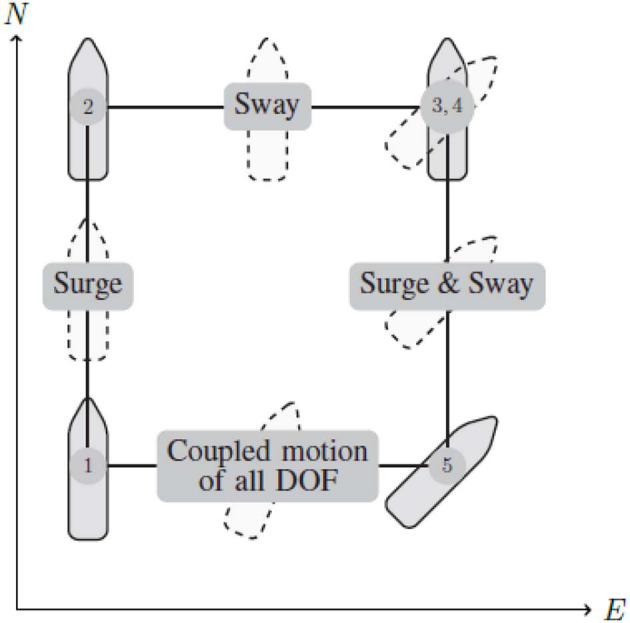
Four corner DP test, for testing trajectory tracking in individual, and coupled degrees of freedom.

**Figure 6:**
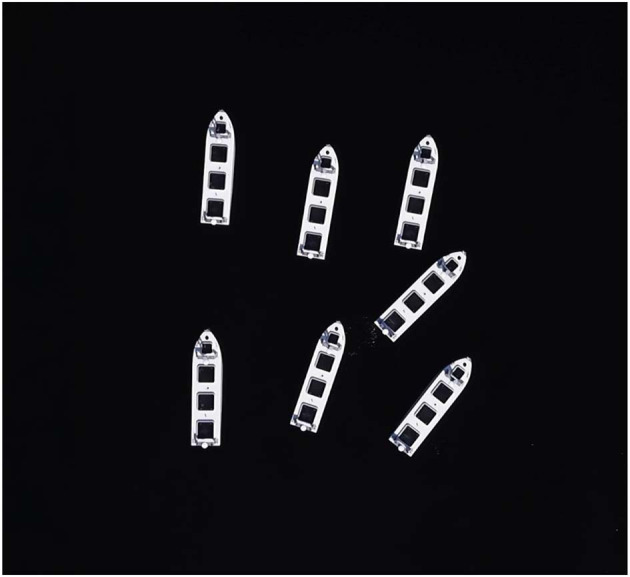
Time-lapse drone photo of four corner DP test. It should be noted that the time-lapse above is of an early test, where errors in the navigation system resulted in poor performance.

In order to test the dynamic positioning capabilities of the control method, the four corner test seen in Figures 5, 6 is used. The four corner DP test is used, as it shows the tracking capabilities of the vessel for individual degrees of freedom, as well as the coupled motion of all degrees of freedom, it is also worth noting that the vessel returns to the initial pose, meaning the test can easily be repeated. The four corner test starts with the vessel pointing north 0°, then performs the following commands:

1. Change position *l* meters due north, and come to a complete stop. This tests the surge motion of the vessel.
2. Change position *l* meters due east, and come to a complete stop. This tests the sway motion of the vessel.
3. Change heading 45°, and come to a complete stop. This tests the yaw motion of the vessel.
4. Change position *l* meters due south, and come to a complete stop. This tests the coupled surge and sway motion of the vessel.
5. Change position *l* meters due west, and heading to 0° and come to a complete stop. This tests coupled motion of all degrees of freedom.

For the box test we chose the box side length *l* to be 5 m, and the reference path was generated by linearly interpolating the pose between the commands, with 55 s to execute each command and a 5 s pause between commands in order for the reference filter to catch up to the reference, and ensure that the vessel comes to a stop. The reference poses used for the experiments are given in Table 2.

**Table 2:** Reference pose for four corner DP test, note that the reference that was used was a linear interpolation of the poses in the table.

**Time **[*s*]****	**0**	**55**	**60**	**115**	**120**	**175**	**180**	**235**	**240**	**295**	**300**
*x*_*r*_ [*m*]	0	5	5	5	5	5	5	0	0	0	0
*y*_*r*_ [*m*]	0	0	0	5	5	5	5	5	5	0	0
ψ_*r*_ [*deg*]	0	0	0	0	0	45	45	45	45	0	0

In order to evaluate the performance of the dynamic positioning, The Integral Absolute Error (IAE) given in (43) was used.

(43) $$\begin{array}{r} IAE\left(t\right)=\int_{0}^{t}\sqrt{{\left(\bar{\eta }-{\bar{\eta }}_{d}\right)}^{⊤}\left(\bar{\eta }-{\bar{\eta }}_{d}\right)}dt \end{array}$$

Where $\bar{\eta }$ and ${\bar{\eta }}_{d}$ are the normalized pose vectors, normalized between ±5 m in north and east direction, and ±50° in heading, giving the following.

$$\begin{array}{l} \bar{\eta }={\left[\frac{x}{5},\frac{y}{5},\frac{\psi }{50}\right]}^{⊤},\;{\bar{\eta }}_{d}={\left[\frac{x_{d}}{5},\frac{y_{d}}{5},\frac{\psi_{d}}{50}\right]}^{⊤} \end{array}$$

**Figure 7:**
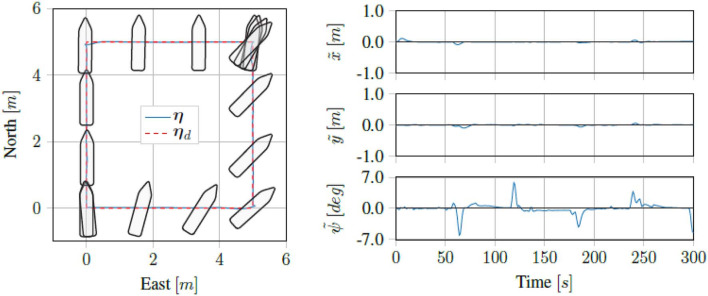
Simulation results for four corner DP tests.

**Figure 8:**
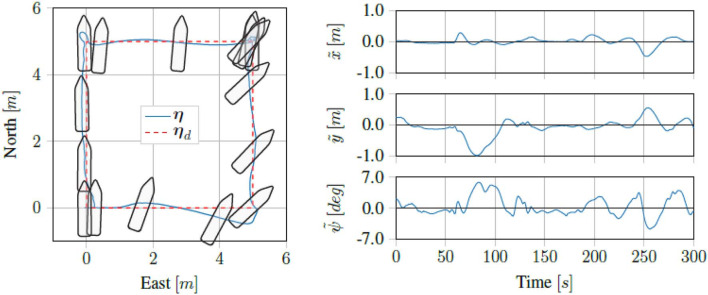
Sea trial results for four corner DP tests.

**Figure 9:**
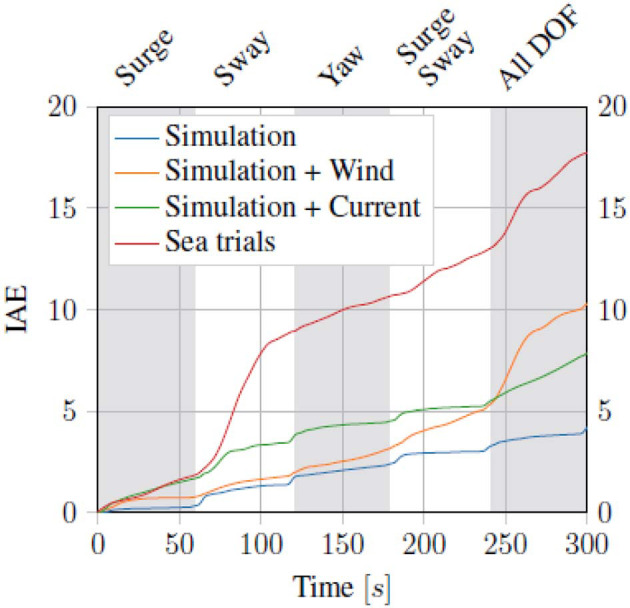
Integral absolute error (IAE) for the dynamic positioning task, the gray and white bands mark the different commands/phases of the four corner test.

Running the proposed control scheme in simulations on the Digital Twin of the *ReVolt* vessel, we got the trajectory and errors seen in Figure 7. For the same test performed on the physical vessel during the sea trials, we got the trajectory and errors seen in Figure 8. The IAE for the tests are shown in Figure 9.

#### 3.3.2. Path Tracking

For both straight line path tracking and curved path tracking, the way-points in Table 3 were used to generate a linearly, and quadratically interpolated path, respectively. For the heading, the path direction was used to generate the desired heading, giving the following reference heading.

(44) $$\begin{array}{r} \psi_{r}=\text{atan}2\left({\overset{∙}{y}}_{r},{\overset{∙}{x}}_{r}\right) \end{array}$$

**Table 3:** Reference pose for the straight line path and curved path, note that the reference that was used was for straight line path tracking was a linear interpolation of the poses, and the reference pose for the curved path, was a quadratic interpolation of the poses.

**Time **[*s*]****	**0**	**100**	**200**	**300**
*x*_*r*_ [*m*]	0	50	100	150
*y*_*r*_ [*m*]	0	0	50	50

In order to encourage the vessel to converge to path in the surge direction, a small cross term was added in the state cost function *Q*(**ζ**) between the heading error, and the position error in the surge direction of the body frame. The key insight here, is that the for large errors in surge, this term will encourage the vessel to turn the bow toward the desired position, meaning the vessel is encouraged to travel in the surge direction, which is the most efficient direction of travel, due to the design of the hull. For our implementation, where pose error is given in the body frame of the vessel, and the state penalty is given as a quadratic function *Q*(**ζ**) = **ζ**^⊤^***Q*ζ**, this penalty is added by simply adding a term to the off-diagonals of ***Q*** corresponding to the cross terms between position error in the *y* direction, and the heading error.

**Figure 10:**
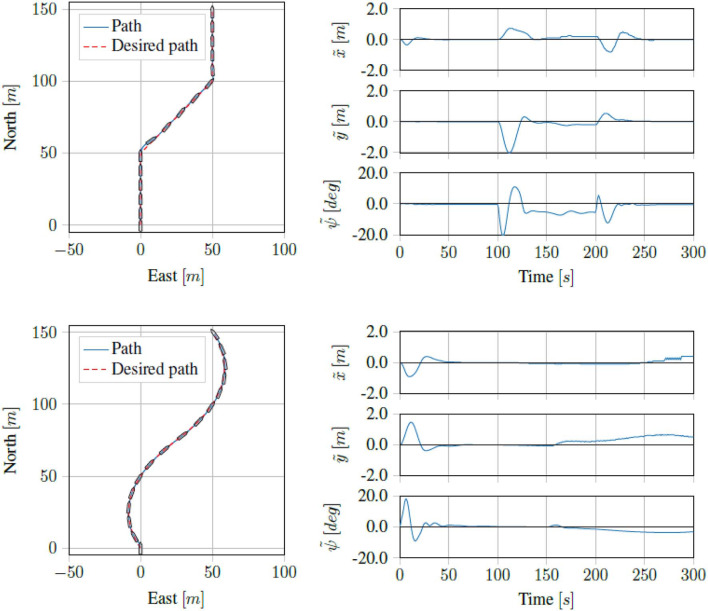
Simulation results for straight line and curved path tracking.

**Figure 11:**
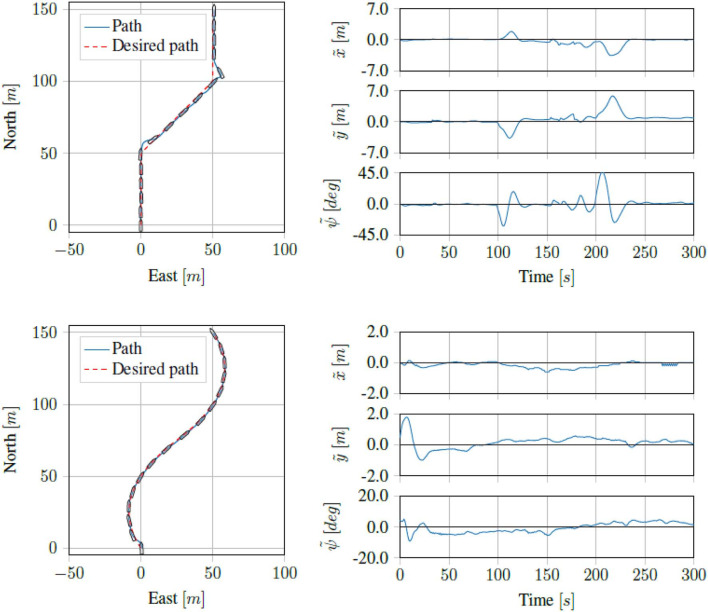
Sea trial results for straight line and curved path tracking.

Running the straight line path tracking on the Digital Twin of the *ReVolt* vessel we got the results seen in Figure 10. Running the same tests on the physical vessel, we got the results seen in Figure 11. As we can see, the proposed control scheme is able to follow the path quite well.

### 3.4. Results

Based on the results, the proposed method seems to work very well, in both simulations and the physical platform. While the simulator has been designed to perform as closely as possible to the physical platform, there are slight discrepancies that may explain the performance drop. The main factors of the performance drop is however most likely due to the measurement and observation noise that is present on the physical vessel. While the RTK GNSS is able to give a good measurement for the pose of the vessel, the estimated vessel velocities that the algorithm is dependent on become very inaccurate, especially at low speeds when the signal to noise ration becomes small. Another error source is likely the thruster dynamics. While the algorithm above assumes the desired thrust is produced immediately, in reality producing the desired thrust vector takes time, as the thrust allocation involves rotating the thrusters to a given angle, as well as spinning up to a desired motor RPM. An additional source of error may also have been a vertical stabilizer, which had recently been added to the vessel between the two rear thrusters, but had not been taken into account in the thrust allocation algorithm. Overall, the results are quite good, especially considering the size of the vessel, the relatively low thrust capability, and the precision to which the maneuvers are performed, even under the uncertainty created by the sensor noise, and environmental forces.

## 4. Assurance of RL-Based Controllers

Assurance is the structured collection of evidence supporting claims and arguments that a system is safe or fit for its intended purpose. Assurance is required to develop trustworthy systems and solutions for use in real-world applications. Principles of assurance can be found in any certification or verification framework, where claims and arguments most often can be considered requirements of verification, while evidence is the result from this verification. Two types of verification are used: (1) *Product verification*, which performs direct verification of the developed product or system and produces *primary evidence*; (2) *process verification*, which performs verification of some part of the development process and produces *circumstantial evidence*. Using established verification frameworks applied to conventional marine control systems, experience has shown what requirements and evidence are most important when verifying these conventional control systems. With novel technology, such as data-driven methods, the verification requirements and evidence that is needed for assurance are still unknown, as they pose a new set of challenges when assuring the system.

Data-driven approaches are not new, but with increasing computational power and abundance of data there has been an increasing interest in these methods. Within control theory, the field of system identification has been a key part of control engineering for many years (Åström et al., 1965; Ho and Kálmán, 1966), and data-driven modeling for control purposes has been practiced since. Such models are typically based on the physical properties that govern the system, and hence the parameters estimated by such methods may reflect measurable properties of the system, thus providing benchmarks for verification. However, most models represent the physical system only within certain operational limits, e.g., weather or sea states, or for certain vessel speed ranges, which restricts the validity of the models accordingly.

Contrary to the more static nature of classical data-driven approaches, where tuning the control parameters relies on offline estimation of the model parameters, in this paper the key difference is that model based-RL is used for online tuning of both the vessel model (including an estimation of unknown disturbances) and the control policy parameters. The vessel model and the control policy are based on proven methods used in the maritime industry for vessel station keeping and guidance, but there are still some key issues that must be considered. For instance, the control policy is highly dependent on an instantaneously valid vessel model, which in turn means the behavior of the vessel is highly dependant on the validity of the learned model. Both the vessel model and the control policy parameters are all continuously learned, but it is critical that all allowed parameter combinations give a sufficiently safe behavior. The proposed control scheme in this paper continuously learns and updates the parameters in order to optimize the tracking behavior. In terms of safety, the main concern is whether the learned model and policy parameters lead to a safe and acceptable behavior. Verifying this in a setting where the parameters are learned online is still an open problem.

Amodei et al. (2016) discusses five basic concrete problem areas related to RL and safety, which must be taken into account for any application of RL.

1. Avoiding reward hacking: the first problem, is that of hacking or gaming the cost function. For the tracking problem in this paper, a positive definite quadratic penalty on the error dynamics is used. From control theory these methods are known to converge to the origin, i.e., where the error is zero. This means the intended behavior is guaranteed when the policy converges to the optimal policy.
2. Avoiding negative side effects: the second problem of avoiding negative side effects, is similar to the first, but addresses the issue of choosing the cost function such that the optimal policy does not give bad or unintended behavior. For the method proposed in this paper, making such guarantees is quite difficult, as tuning the parameters of the quadratic cost function will still have an effect on the vessel behavior when converging to the origin. One example of this is that we typically want the vessel to approach the path head on if we have a large deviation between the position we are at, and the desired position. Tuning the parameters of cost function in order to get this behavior is not trivial.
3. Scalable oversight: this pertains to how we can ensure that the RL agent respects aspects of the objective that are encountered infrequently. In terms of the trajectory tracking problem, the environment is quite limited, and the objective is clearly defined, hence the problem of scalable oversight is of limited relevance to the work presented in this paper.
4. Safe exploration: exploration is necessary in order to improve performance, but bears risk, and thus performing exploration in a safe manner is not trivial. Safe exploration also encompasses the evaluation of the quality of the training data that is gathered. For a real world application, this means accounting for faulty hardware, and noisy measurements, which may lead to problematic training data. For the method proposed in this paper, where the system is learning continuously online, the problem of safe exploration and learning is highly relevant. Some measures are taken, such as using batches of training data and restricting the values that the policy parameterization may take. However, these measures only serve to mitigate potential problems, hence safe exploration and learning is still an open problem.
5. Robustness to distributional shift: this refers to how we can ensure the agent is robust to changes in the operating environment. For the proposed method, this is mostly solved by continuously learning online, which ensures that the agent learns the distributional shift when the environment changes. However, as discussed in the fourth problem of safe exploration, learning online complicates the matter of ensuring data quality.

In order to produce the evidence needed for verification of data driven methods, there are two main approaches, namely *scenario based verification*, and *theoretical verification*. *Scenario based verification* would be to conduct extensive testing in representative scenarios, which in practice would mean simulation-based testing as this would be the only feasible online solution. More limited real testing should also be used, but targeted toward validating the simulation accuracy. Many RL solutions are in practice not viable without simulation-based training or development and this would mean the same tool can be utilized both for testing, and offline training. The challenge in this case would be to induce a representative set of scenarios to prove the safety or validity of the solution, and such scenario selection is an open question for testing AI or systems operating in complex environments in general. The second approach *theoretical verification*, would be to impose constraints on the RL algorithm in order to avoid unwanted behavior. This would entail combining methods from control theory, a physical or mathematical understanding of the system, and experience or insights of the control scheme, in order to express and implement various constraints on the learnable model and policy parameters. This may not conceptually be a new approach since similar methods already exist, but this approach is difficult to use in practice, as finding parameter constraints that ensure safe operations is non-trivial.

In conclusion, an assurance framework for technologies, such as the one presented in this paper is an open research question. However, one can with confidence state that it should include both process and product verification, i.e., considering not only what is developed, but also how it is developed. This would mean that adequate development and assurance processes should be developed, including verification methods that can produce the required evidence both for efficient development, as well as assurance. In addition, novel data-driven methods should be combined with prior knowledge, verified solutions and proven physical or mathematical relations (Eldevik, 2018). This, in order to be able to explain the behavior, and in turn guarantee that the methods are safe and fit for the intended purpose.

## 5. Conclusion

The proposed method performed very well in all three tested tracking scenarios both in simulations and in sea trials. The method is also versatile, as using it on different vessels only requires knowledge of the inertia matrix, with the update laws providing a tool for learning the other model parameters, and a control policy. For future work, it may be interesting to improve and update the thrust allocation algorithm to get a smaller error between produced and desired thrust, and investigate whether this results in better accuracy. Alternatively, feedback from the thrusters can be used to get better estimates of the thrust vector for use in the data stack, and model estimation. For the value function, polynomial basis functions were used, and the estimator was linear in the parameters, which leads to a limited estimation capability. Deep learning methods could lead to more accurate value function approximation, albeit at the expense of transparency and interpretability. Procedures for implementing online assurance would add great value to current research practices. One possible way to do this is by using a new parameterization of the control law once it has been verified, either by simulation, or by constraining the parameterization to a set of parameters that is known to be safe.

## Data Availability Statement

The raw data supporting the conclusions of this article will be made available by the authors, without undue reservation, to any qualified researcher.

## Author Contributions

AM and AL conceived the presented idea. AM developed the theory and performed the simulations, while AM, TP, and AL carried out the experiments. JG and TP contributed with the assurance section from a classification society perspective. AM, AL, and SG discussed the results and methods. All authors contributed to the final manuscript.

### Conflict of Interest

JG and TP were employed by the company DNV GL. The remaining authors declare that the research was conducted in the absence of any commercial or financial relationships that could be construed as a potential conflict of interest.
